# Differential Effects of Prenatal Poly I:C Exposure and Antipsychotics on NMDA/GABA Receptors and GSK3β‐Mediated Signaling in the Dorsal Raphe Nucleus of Female Rats

**DOI:** 10.1111/fcp.70033

**Published:** 2025-06-25

**Authors:** Shiyan Chen, Jiamei Lian, Yueqing Su, Chao Deng

**Affiliations:** ^1^ Department of Neurology The First Affiliated Hospital of Fujian Medical University, and The Binhai Campus of Fujian Medical University First Hospital, National Regional Medical Center Fuzhou China; ^2^ School of Medical, Indigenous and Health Sciences, and Molecular Horizons University of Wollongong Wollongong NSW Australia; ^3^ Fujian Maternity and Child Health Hospital, College of Clinical Medicine for Obstetrics & Gynaecology and Paediatrics Fujian Medical University Fuzhou China

**Keywords:** AKT‐GSK3β, antipsychotics, dorsal raphe nucleus, maternal immune activation, NMDA, PKA

## Abstract

**Background:**

The dorsal raphe nucleus (DRN) is the origin of the 5‐HT neurotransmission pathways. The 5‐HT, dopamine D2, GABA, and NMDA receptors, as well as the cyclic adenosine monophosphate (cAMP)‐protein kinase A (PKA) and G protein‐independent protein kinase B (PKB/Akt)‐glycogen synthase kinase 3β (GSK3β) signaling, are involved in the pathophysiology of schizophrenia and are modulated by antipsychotics. However, their pathological changes and antipsychotic modulations in the DRN are not well understood in schizophrenia.

**Objectives:**

This study explored effects of antipsychotics on NMDA and GABA_A_ receptors, as well as PKA, AKT‐GSK3β, cAMP‐responsive element‐binding protein 1 (CREB1), and disheveled (Dvl)‐β‐catenin signaling in the DRN using a maternal immune activation rat model.

**Methods:**

Prenatal polyriboinosinic‐polyribocytidylic acid (Poly I:C) exposure was delivered at gestational Day 15. Female rats were treated with risperidone, olanzapine, or vehicle from postnatal day 70 for 35 days.

**Results:**

Prenatal Poly I:C exposure increased mRNA expression of NMDA receptor *Grin2a/2b* subunits, the GABA_A_ receptor *β3* subunit, glutamic acid decarboxylase 1 (*GAD1*), *AKT1/3*, and *GSK3β* in the DRN. Antipsychotics significantly increased the mRNA expression of *PKA*, *CREB1*, *β‐catenin*, *GSK3β*, and *Grin2d* subunits in the DRN of Poly I:C rats. Prenatal Poly I:C exposure led to decreased expression of *GAD2*, which was partially reversed antipsychotics.

**Conclusion:**

This study suggests that prenatal Poly I:C exposure and antipsychotics differentially modulate NMDA and GABA_A_ receptors, as well as AKT‐GSK3β, PKA‐CREB1, and Dvl‐β‐catenin signaling in the DRN of rats. Poly I:C mainly influenced the AKT‐GSK3β signaling, while antipsychotics modulated the AKT‐GSK3β, PKA‐CREB1, and Dvl‐GSK3β‐β‐catenin signaling pathways in the DRN.

**Abbreviations**
5‐HT: serotoninergic
ANOVA: analysis of variance
b.i.d.: bis in die (twice a day)
cAMP: cyclic adenosine monophosphate
CREB1: cAMP‐responsive element‐binding protein 1
D2R: dopamine D2 receptor
DRN: dorsal raphe nucleus
Dvl: disheveled
GABA: γ‐aminobutyric acid
GAD: glutamic acid decarboxylase
GD: gestational day
GSK3β: glycogen synthase kinase 3β
IP: intraperitoneally
NMDA: glutamatergic *N*‐methyl‐D‐aspartate
PKB/Akt: protein kinase B
PKA: protein kinase A
Poly I:C: polyriboinosinic‐polyribocytidylic acid
qRT‐PCR: quantitative real‐time polymerase chain reaction
TLR3: toll‐like receptor 3

## 1 Introduction

Epidemiological and experimental evidence implicates gestational infections as one important factor in the pathogenesis of neuropsychiatric disorders, such as schizophrenia [1]. Maternal immune activation (MIA) during pregnancy increases the risk of the offspring developing neuropsychiatric disorders, which could cause behavioral abnormalities and brain changes in the offspring whose mothers were exposed to MIA, via altered neuroimmune modulations [1, 2]. One common approach to induce MIA in rats is to administer the viral mimetic synthetic double‐stranded RNA analogue polyriboinosinic‐polyribocytidylic acid (Poly I:C) to pregnant animals [3, 4]. Consistent with postmortem studies in schizophrenia, prenatal Poly I:C exposure also caused neurotransmission deficits in dopaminergic, serotoninergic (5‐HT), glutamatergic *N*‐methyl‐D‐aspartate (NMDA), and γ‐Aminobutyric acid (GABA) receptors in the prefrontal cortex, hippocampus, nucleus accumbens, and caudate putamen [2, 5, 6, 7].

Monoaminergic neurotransmitter systems, originating in the brain stem and innervating widespread brain regions, have been the focus of various theories of the etiology and treatment of schizophrenia [7, 8, 9, 10]. The brain stem, origins of dopamine, noradrenaline, acetylcholine, and 5‐HT neurotransmission systems, has attracted attention as a possible pathological site of schizophrenia [9, 11, 12, 13, 14]. The cell bodies of 5‐HT‐containing neurons are situated in the raphe nuclei of the brain stem; the principal source of ascending projections is the dorsal raphe nuclei (DRN), which innervate the majority of forebrain regions [15, 16]. Although there is evidence of a dysconnectivity of the DRN and forebrain regions in schizophrenia [17], few studies have explored the role of the DRN in the pathophysiology of schizophrenia and the actions of antipsychotic drugs, possibly due to the small size of the nucleus and the difficulty of sampling.

One major target of antipsychotic drugs is the dopamine D2 receptors (D2R) [18, 19]. There are two major D2R downstream cellular signaling pathways: the G protein‐dependent cyclic adenosine monophosphate (cAMP)‐protein kinase A (PKA) pathway, and the G protein‐independent protein kinase B (PKB/Akt)‐glycogen synthase kinase‐3 beta (GSK3β) pathway [20]. Both cAMP‐PKA and AKT‐GSK3β signaling pathways were involved in the pathophysiology of schizophrenia and the actions of antipsychotics [21, 22, 23, 24, 25, 26, 27, 28]. Through them, antipsychotics modulate several other pathways or substrates, such as the disheveled (Dvl)‐GSK3β‐β‐catenin pathway and cAMP‐responsive element‐binding protein 1 (CREB1) [24, 25, 27, 29, 30]. Therefore, this study investigated the effect of antipsychotic drugs (olanzapine and risperidone) on the expression of 5‐HT2, D2, GABA_A_, and NMDA receptors, as well as the PKA, AKT‐GSK3β, CREB1, and Dvl‐β‐catenin signaling in the DRN using a female Poly I:C rat model.

## 2 Materials and Methods

### 2.1 Animals and Prenatal Poly I:C Treatment

Time‐mated pregnant Sprague–Dawley rats (gestational day (GD) 8) were bought from the Animal Resource Centre, Perth, Australia. They were housed individually in Techniplast GR1800 double‐decker rat ventilated cages and allowed to habituate to their surroundings for 1 week. At GD15, the pregnant rats were injected intraperitoneally (IP) with either Poly I:C (InvivoGen, Toulouse, France, 5 mg/kg, dissolved in 1% saline, *n* = 7), which has been successfully used to generate Poly I:C psychiatric rat models, or an equivalent volume of saline (*n* = 7) [31, 32, 33]. The day of birth was considered as postnatal day (PD) 0. Newborn rats were housed with the mother till weaning on PD21, and then only females were used in this study. After weaning, rats were housed in IVCs with a divider under environmentally controlled conditions (22°C; light cycle from 07:00 to 19:00 and dark from 19:00 to 07:00) with *ad libitum* access to water and standard laboratory chow diet (3.9 kcal/g: 10% fat, 74% carbohydrate, 16% protein) throughout the experimental period. Each cage housed two rats from the same treatment group, and the divider (with perforated holes to allow the two rats to see, hear, and smell each other) separated the cage into two chambers of equal size with their own enrichment devices, including a plastic tunnel, a wood stick, and nesting materials with corncob bedding.

### 2.2 Antipsychotic Treatment and Brain Tissue Collection

During PD67–69, female rats were offered cookie dough pellets (0.3 g; 15% gelatin, 9% milk powder, 38% corn flour, and 38% sugar) without drug for training in oral drug delivery. In both healthy (saline) and Poly I:C cohorts, rats were orally administered twice per day (b.i.d.) vehicle (0.3‐g cookie pellets as controls, *n* = 11), risperidone (0.9 mg/kg, Janssen, Italy, *n* = 12), olanzapine (3 mg/kg, Eli Lilly, USA, *n* = 11) from PD70 for 35 days following the methods routinely used in our laboratory [34, 35]. The clinically used drug tablets were ground into powder. After calculating drug dosage based on the body weight of rats, the weighed powder was mixed with 0.3‐g cookie dough and droplets of water prior to administration. The pellets with or without drugs were then offered to rats on a metal spoon and observed to ensure complete consumption by the rats. Two hours after the final treatment, rats were euthanized by isoflurane anesthesia and decapitation. The collected brains were frozen in liquid nitrogen and stored at −80°C. The discrete brain regions were collected using a brain microdissection puncture technique in a Cryostat (at −10.5°C ± 1.5°C) as previously reported [34, 36]. According to the rat brain atlas [37], the brain tissues through the DRN (Bregma −7.04 to −9.30 mm) were dissected and kept at −80°C for future use.

### 2.3 RNA Isolation and Gene Expression Analysis by Real Time qPCR

Total RNA from the DRN brain tissue was prepared using the PureLink RNA Mini Kit (#12183025; Invitrogen Life Technologies, Carlsbad, CA, USA). cDNA was synthesized from purified RNA using the High‐Capacity cDNA Reverse Transcription Kits (#4368814; Thermo Fisher Scientific, Waltham, MA, USA). qRT‐PCR was performed in duplicate on a Quant Studio qRT‐PCR system (Thermo Fisher, Waltham, MA, USA) using TaqMan Gene Expression Assays (Life Technologies, Sydney, NSW, Australia) for *β‐actin* (Hs01060665_g1), *Gapdh* (Rn01775763_g1), *Gabrb1* (Rn00564146_m1), *Gabrb2* (Rn00564149_m1), *Gad2* (Rn00561244_m1), *Grin2c* (Rn00561359_m1), and *Grin2d* (Rn00575638_m1). SYBR Green PCR Master Mix (Life Technologies, Sydney, NSW, Australia) was used for *Htr1a*, *Htr2a*, *Htr2c*, *Drd2*, *Gabrb3*, *Gad1*, *Grin1*, *Grin2a*, *Grin2b*, *Akt1*, *Akt2*, *Akt3*, *Gsk3β*, *Prkaca*, *Prkacb*, *Creb1*, *Ctnbb1*, and *Dvl3*, (Primers details see Table S1). The cycling parameters were 95°C for 10 min followed by 40 cycles (95°C 15 s, 60°C 1 min). Target gene relative expression levels were normalized to two house‐keeping genes (*β‐Actin* and *Gapdh*). The 2^−ΔΔCT^ method was used to calculate the results.

### 2.4 Statistical Analysis

SPSS software (version 21.0, IBM, NY, USA) was used to analyze all data. The outliers were identified and removed using Boxplot. The Shapiro–Wilk test was used to examine the data distribution. Data were analyzed by a two‐way ANOVA (Poly I:C × Antipsychotics). Post hoc Fisher's least significant difference multiple comparisons tests were followed for comparison between groups. All data are expressed as mean ± SEM, and statistical significance accepted when *p* < 0.05.

## 3 Results

### 3.1 Effects on the Expression of Neurotransmitter Receptors

#### 3.1.1 NMDA Receptors

**FIGURE 1:**
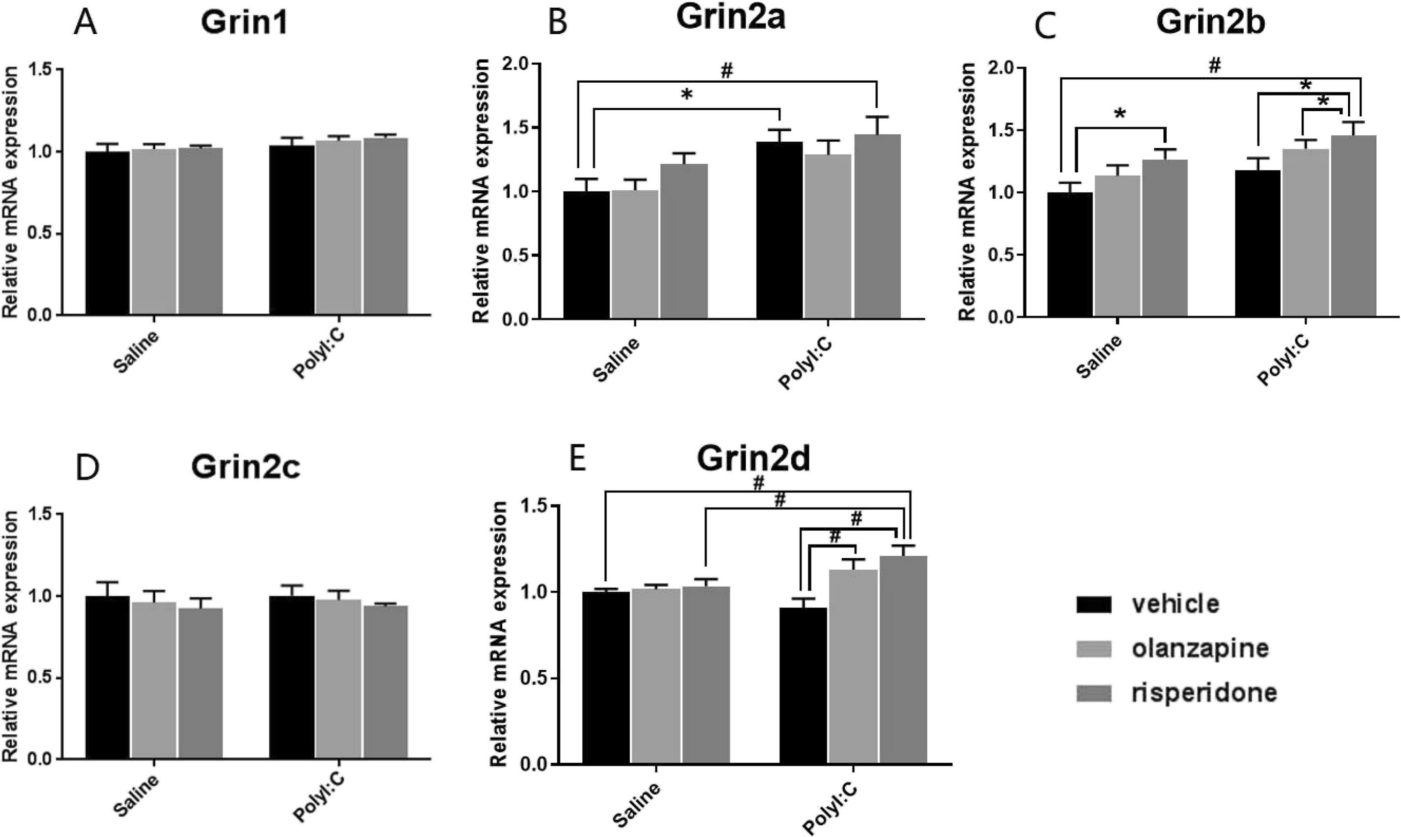
The effect of prenatal Poly I:C exposure and adult antipsychotic (risperidone and olanzapine) treatment on the mRNA expression of (A) NMDA receptor 1 (*Grin1*) subunit, (B) NMDA receptor 2A (*Grin2a*) subunit, (C) NMDA receptor 2B (*Grin2b*) subunit, (D) NMDA receptor 2C (*Grin2c*) subunit, and (E) NMDA receptor 2D (*Grin2d*) subunit in the dorsal raphe nuclei of female rats. Data were presented as mean ± SEM, * *p* < 0.05, # *p* < 0.01.

There was a significant main effect of Poly I:C (F_1, 28_ = 12.100, *p* = 0.002), but no effect of antipsychotics on *Grin2a* mRNA expression (F_2, 28_ = 1.646, *p* = 0.211); there were also no significant interactions between the two factors (F_2, 28_ = 0.303, *p* = 0.741) (Figure 1B). Post hoc comparisons showed significantly higher *Grin2a* mRNA levels in Poly I:C‐vehicle offspring (*p* = 0.015, Figure 1B) and Poly I:C‐Risperidone offspring (*p* = 0.004, Figure 1B) than in Saline‐Vehicle rats. *Grin2b* mRNA expression levels were significantly affected by the Poly I:C factor (F_1, 28_ = 7.228, *p* = 0.012) and the antipsychotics factor (F_2, 28_ = 4.956, *p* = 0.014) (Figure 1C). Risperidone significantly increased *Grin2b* mRNA levels (Poly I:C‐Risperidone vs. Saline‐Vehicle, *p* = 0.001; Poly I:C‐Risperidone vs. Poly I:C‐Vehicle group, *p* = 0.033; Saline‐Risperidone vs. Saline‐Vehicle, *p* = 0.035; Poly I:C‐Risperidone vs. Saline‐Olanzapine, *p* = 0.012; Figure 1C). There was also a significant main effect of the antipsychotics factor (F_2, 28_ = 7.433, *p* = 0.003) and a significant interaction between the Poly I:C and antipsychotics factors (F_2, 28_ = 4.876, *p* = 0.015) on the *Grin2d* mRNA expression, but without a significant effect of the Poly I:C factor (F_1, 28_ = 3.172, *p* = 0.086; Figure 1E). Antipsychotics (olanzapine and risperidone) increased *Grin2d* mRNA levels in Poly I:C offspring (Poly I:C‐Olanzapine vs. Poly I:C‐Vehicle, *p* = 0.003; Poly I:C‐Risperidone vs. Poly I:C‐Vehicle, *p* < 0.001; Poly I:C‐Risperidone vs. Saline‐Vehicle, *p* = 0.002; Poly I:C‐Risperidone vs. Saline‐Risperidone, *p* = 0.008; Figure 1E). However, there were no significant effects of the Poly I:C or antipsychotic factor on *Grin1 and Grin2c* mRNA expression (all *p* > 0.05, Figure 1A,D).

#### 3.1.2 GABAergic Markers

**FIGURE 2:**
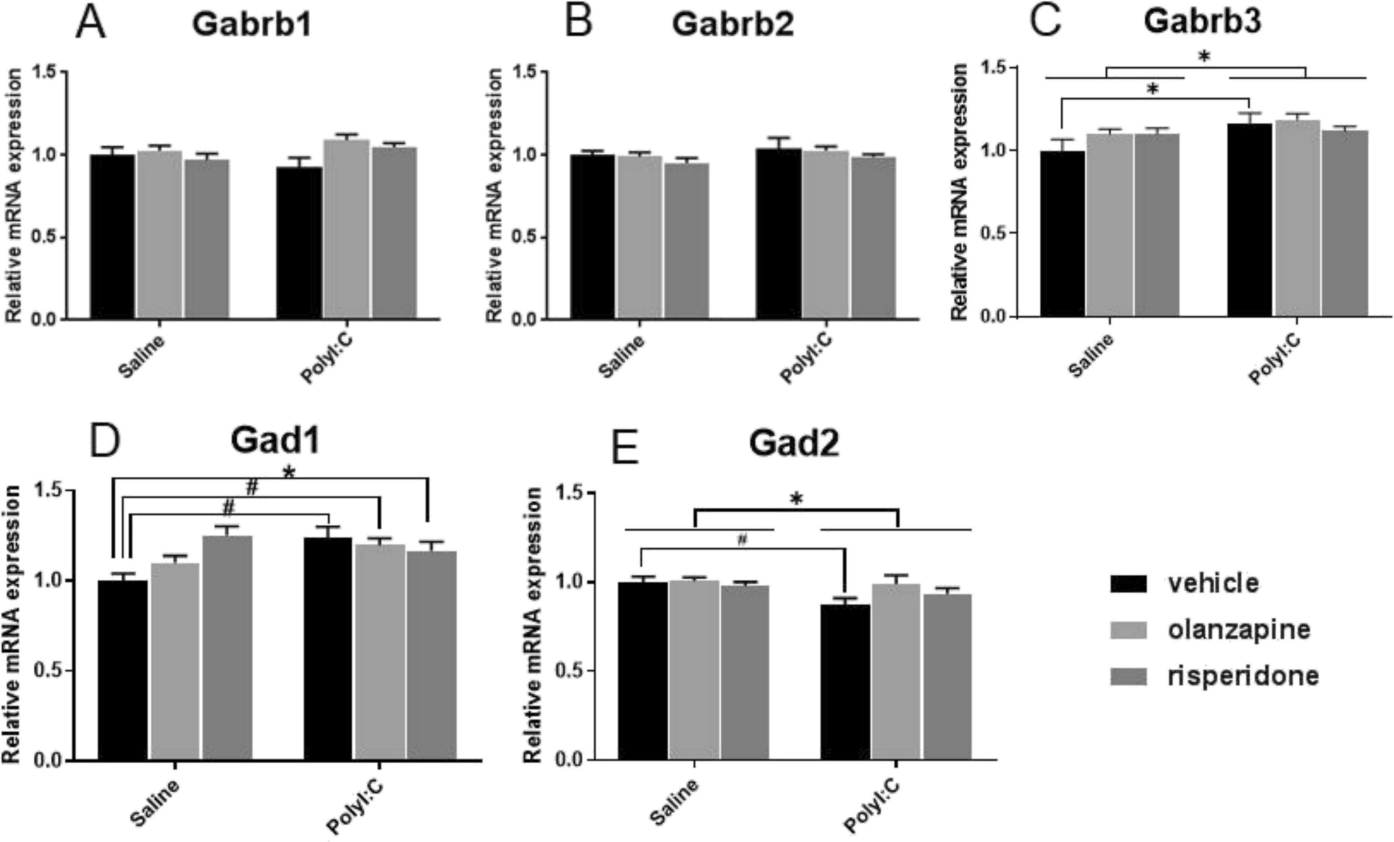
The effect of prenatal Poly I:C exposure and adult antipsychotic (risperidone and olanzapine) treatment on the mRNA expression of (A) GABA_A_ receptor β1(*Gabrb1*) subunit, (B) GABA_A_ receptor β2 (*Gabrb2*) subunit, (C) GABA_A_ receptor β3 (*Gabrb3*) subunit, (D) glutamic acid decarboxylase *GAD1*, and (E) glutamic acid decarboxylase *GAD2* in the dorsal raphe nuclei of female rats. Data were presented as mean ± SEM, * *p* < 0.05, # *p* < 0.01.

There was a significant effect of Poly I:C factor on *Gabrb3* mRNA expression (F_1, 28_ = 5.673, *p* = 0.024; Figure 2C). The mRNA expression of *Gabrb3* was significantly higher in the Poly I:C‐Vehicle than in the Saline‐Vehicle groups (*p* = 0.019, Figure 2C). For the primary GABA‐synthesizing enzymes, there was a significant main effect of Poly I:C factor (F_1, 28_ = 4.626, *p* = 0.040) and a significant interaction between Poly I:C and antipsychotics factors (F_2, 28_ = 5.890, *p* = 0.007) on *Gad1* mRNA expression. Post hoc comparisons showed significantly higher *Gad1* mRNA levels in the Poly I:C‐Vehicle (*p* = 0.002), Poly I:C‐Olanzapine (*p* = 0.008), and Poly I:C‐Risperidone (*p* = 0.016) groups than those in the Saline‐Vehicle rats (Figure 2D). Conversely, prenatal Poly I:C exposure significantly decreased *Gad2* mRNA levels of Poly I:C offspring compared with the control (F_1, 28_ = 5.882, *p* = 0.022; Figure 2E). However, no significant difference was observed in the mRNA levels of *Gabrb1* and *Gabrb2* in rats treated with Poly I:C or antipsychotics compared with the control (all *p* > 0.05, Figure 2A,B).

There were no significant effects of Poly I:C or antipsychotic factor on *Drd2*, *Htr1a*, *Htr2a*, *and Htr2c* mRNA expression, and no significant interaction between the two factors (all *p* > 0.05).

### 3.2 Effects on Akt‐GSK3β Signaling

**FIGURE 3:**
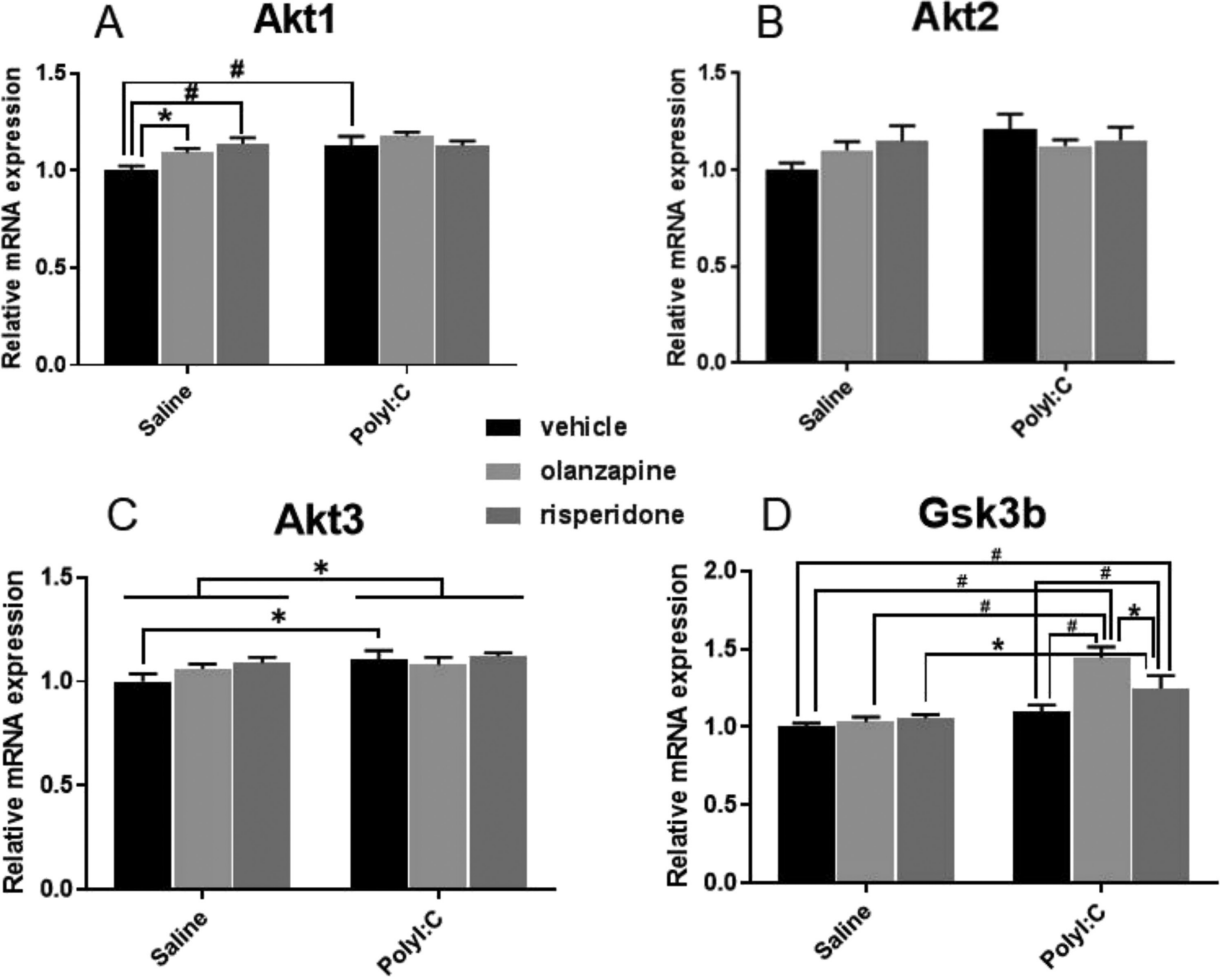
The effect of prenatal Poly I:C exposure and adult antipsychotic (risperidone and olanzapine) treatment on the mRNA expression of (A) Akt1, (B) Akt2, (C) Akt 3, and (D) GSK3β in the dorsal raphe nuclei of female rats. Data were presented as mean ± SEM, * *p* < 0.05, # *p* < 0.01.

There were significant main effects of Poly I:C (F_1, 28_ = 7.212, *p* = 0.012) and antipsychotic drugs factors (F_2, 28_ = 3.582, *p* = 0.0412) on *Akt1* mRNA expression, but without a significant interaction between the two factors (F_*2, 28*_ = 2.690, *p* = 0.085). Antipsychotics significantly increased *Akt1* mRNA levels (Saline‐Olanzapine vs. Saline‐Vehicle, *p* = 0.036; Saline‐Risperidone vs. Saline‐Vehicle, *p* = 0.003; Figure 3A), and prenatal Poly I:C exposure also increased *Akt1* mRNA expression in the DRN of offspring rats (Poly I:C‐Vehicle vs. Saline‐Vehicle, *p* = 0.008; Poly I:C‐Olanzapine vs. Saline‐Vehicle, *p* < 0.001; Poly I:C‐Risperidone vs. Saline‐Vehicle, *p* = 0.005; Figure 3A). There was also a significant effect of the Poly I:C factor (F_1, 28_ = 4.427, *p* = 0.04; Figure 3C), with an increased *Akt3* mRNA expression in the DRN of offspring rats (Poly I:C‐Vehicle vs. Saline‐Vehicle, *p* = 0.023). No significant difference in *Akt2* mRNA expression was observed in the DRN of Poly I:C or antipsychotic treated rats (*p* > 0.05, Figure 3B).

The expression of *Gsk3β* was significantly affected by prenatal Poly I:C exposure (F_1, 28_ = 31.97, *p* < 0.001) or antipsychotics factor (F_2, 28_ = 7.001, *p* = 0.0034); there was also a significant interaction between the two factors (F_2, 28_ = 5.129, *p* = 0.0126; Figure 3D). The *Gsk3β* mRNA level was significantly increased in the Poly I:C‐Olanzapine group (Poly I:C‐Olanzapine vs. Saline‐Vehicle, *p* < 0.001, Poly I:C‐Olanzapine vs. Saline‐Olanzapine, *p* < 0.001, Poly I:C‐Olanzapine vs. Poly I:C‐Vehicle, *p* < 0.001; Figure 3D), and a similar higher *Gsk3β* mRNA level was also observed in the Poly I:C‐Risperidone group (Poly I:C‐Risperidone vs. Saline‐Vehicle, *p* = 0.001, Poly I:C‐Risperidone vs. Saline‐Risperidone, *p* = 0.010, Poly I:C‐Risperidone vs. Poly I:C‐Vehicle, *p* < 0.001; Figure 3D).

### 3.3 Effects on PKA, Creb1, Ctnnb1, and Dvl‐3 Signaling

**FIGURE 4:**
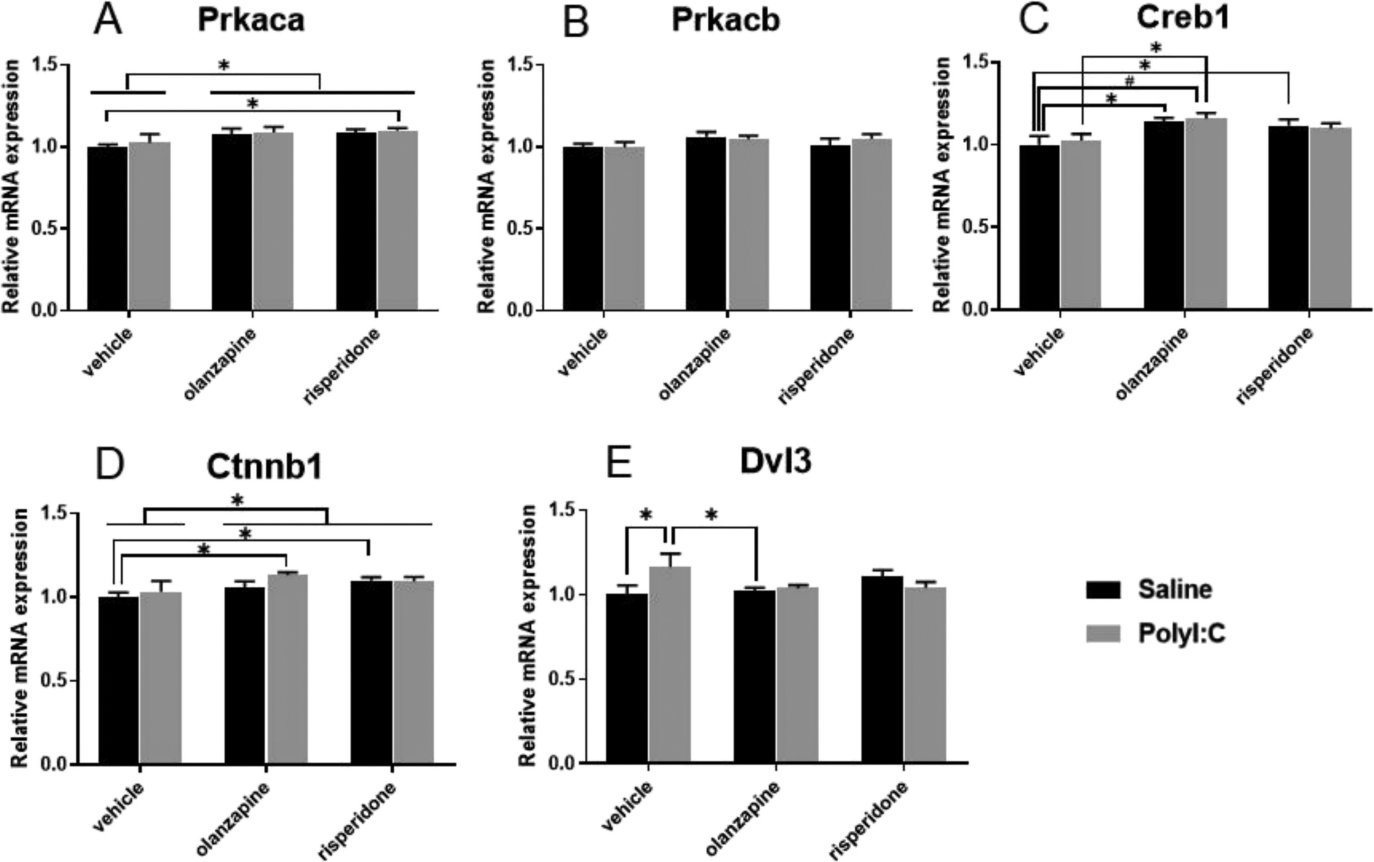
The effect of prenatal Poly I:C exposure and adult antipsychotic (risperidone and olanzapine) treatment on the mRNA expression of (A) Prkaca, (B) Prkacb, (C) Creb1, (D) Ctnnb1, and (E) Dvl3 in the dorsal raphe nuclei of female rats. Data were presented as mean ± SEM, * *p* < 0.05, # *p* < 0.01.

The two‐way ANOVA showed an overall effect of antipsychotics on *Prkaca* mRNA expression (F_2, 28_ = 3.893, *p* = 0.032; Figure 4A), but no effect of Poly I:C (F1, 28 = 0.519, *p* = 0.4773) and interactions between the two factors (F_2, 28_ = 0.008, *p* = 0.992). There were no significant effects of Poly I:C and antipsychotics on *Prkacb* mRNA expression (*p* > 0.05, Figure 4B). There were significant main effects of antipsychotics on the *Creb1* mRNA expression (F_2, 28_ = 6.274, *p* = 0.006; Figure 4C). Both olanzapine and risperidone, respectively, increased *Creb1* mRNA expression (Saline‐Olanzapine vs. Saline‐Vehicle, *p* = 0.011; Saline‐Risperidone vs. Saline‐Vehicle, *p* = 0.0332; Figure 4C).

There was an overall effect of antipsychotics on *Ctnnb1* (F_2_, _28_ = 3.630, *p* = 0.040; Figure 4D), in which *Ctnnb1* mRNA expression was higher in the Saline‐Risperidone than in the Saline‐Vehicle group (*p* = 0.041). There were no main effects of prenatal Poly I:C (F_1, 28_ = 1.080, *p* = 0.308) or antipsychotics factor (F_2, 28_ = 0.776, *p* = 0.470) on *Dvl3* mRNA expression, but a significant interaction between the two factors (F_2, 28_ = 3.548, *p* = 0.0423; Figure 4E). Post hoc comparisons showed a higher *Dvl3* mRNA level in the Poly I:C‐Vehicle than in the Saline‐Vehicle (*p* = 0.016) and Saline‐Olanzapine groups (*p* = 0.033; Figure 4E).

## 4 Discussion

**FIGURE 5:**
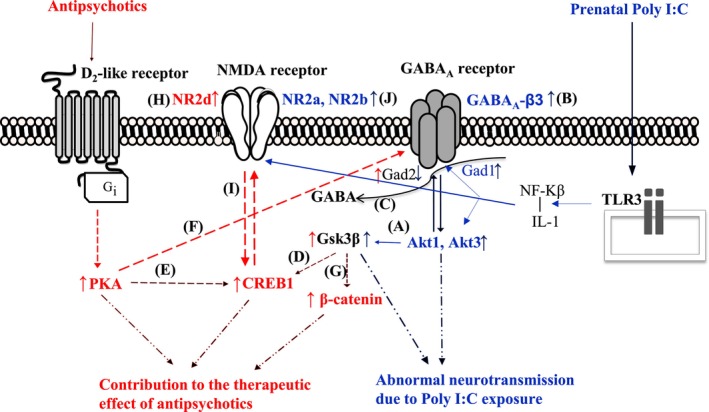
**Interactive effects of prenatal Poly I:C exposure and antipsychotics on various signaling pathways and receptors in the dorsal raphe nuclei.** Our previous study showed that prenatal Poly I:C exposure caused elevation of NF‐κB and IL‐1β inflammatory signaling via activating toll‐like receptor 3 (TLR3) in the brain of offspring [2]. Prenatal Poly I:C exposure increases the expression of Akt and GSK3β (A) and GABAA (β‐3) receptors (B). It also influences GABA production by increasing GAD1 and decreasing GAD2 expression, while antipsychotics may counteract the decrease in GAD2 caused by prenatal Poly I:C exposure (C). Additionally, antipsychotics enhance CREB1 expression, likely through the activation of GSK3β (D) and PKA (E). They may also modulate GABA_A_ receptors via the PKA signaling pathway (F). Furthermore, antipsychotics increase the expression of GSK3β and β‐catenin (G). They elevate the expression of NMDA (NR‐2d) receptors (H), which may further affect CREB1 activity (I). Prenatal Poly I:C exposure also increases the expression of NMDA (NR‐2a, 2b) receptors (J). Abbreviations: IL‐1, interleukin‐1; NF‐Kβ, nuclear factor kappa‐light‐chain‐enhancer of activated B cells; TLR3, toll‐like receptor 3. Red color indicating antipsychotic effects and blue color indicating effects of prenatal Poly I:C exposure.

Previous studies have reported that MIA caused pathological changes in various neurotransmitters (including the dopaminergic, serotonergic, glutamatergic, and GABAergic neurotransmitters). MIA also caused pathological changes in related cellular signaling pathways in various brain regions, including the nucleus accumbens, caudate putamen, hippocampus, prefrontal cortex, and ventral tegmental area [2, 5, 34, 38]. This is the first study to provide evidence that Poly I:C‐elicited MIA and antipsychotics differentially modulate NMDA and GABA_A_ receptors, as well as AKT‐GSK3β, PKA‐CREB1, and Dvl‐β‐catenin signaling in the DRN of female rats (Figure 5).

NMDA receptors are heteromeric, integral membrane proteins formed by the assembly of the obligatory NR1 subunit together with the four different types of modulatory NR2 subunits, NR2A‐NR2D [39, 40]. Various studies have suggested that activation of the immune system can interfere with NMDAR function during brain development, in which abnormal transcript and protein expression of NMDA receptor subunits have been observed in the cortical and hippocampus regions of MIA models [5, 41, 42]. In this study, we have extended the previous findings to also evidence an increased mRNA expression of the NR2A and NR2B subunits in the DRN of Poly I:C adult offspring. Olanzapine and risperidone increased the mRNA level of the NR2D subunit but decreased that of the NR2C subunit in the DRN in this study. Previous studies from our laboratory and others have reported that antipsychotic administration (e.g., clozapine, aripiprazole, haloperidol, olanzapine, and risperidone) could upregulate NMDA receptor binding density and expression of NR1 and NR2A subunits in various brain regions including the nucleus accumbens, hippocampus, and cortex [43, 44, 45, 46], while aripiprazole and haloperidol decreased the expression of these subunits in the ventral midbrain of rats [26]. These findings suggested differential brain regional effects of antipsychotic modulations on NMDA receptors.

GABAergic neurotransmission has been reported to be involved in various mental disorders, including schizophrenia [9, 47], and antipsychotics can regulate GABAergic neurotransmission [28, 44, 48]. Increased binding density of GABA_A_ receptors has been found in the prefrontal cortex, cingulate cortex, superior temporal gyrus, and hippocampus of schizophrenic subjects [49, 50, 51]. Prenatal Poly I:C exposure has been reported to cause abnormal expression of GABA_A_ receptor subunits and GAD1/GAD2 in the prefrontal cortex, hippocampus, nucleus accumbens, and ventral tegmental area of rodent brains [2, 5, 34, 52]. This study provided novel evidence to extend these findings, showing that prenatal Poly I:C significantly increased expression of *Gabrb3* and *GAD1* mRNA but decreased *GAD2* mRNA expression in the DRN of rats.

The AKT‐GSK3β signaling pathway is a noncanonical D2 receptor transduction pathway that has been implicated in the pathophysiology of dopamine‐associated neuropsychiatric diseases such as schizophrenia, bipolar disorder, and depression [23, 53, 54, 55]. A deficit in AKT‐GSK3β signaling was observed in the prefrontal cortex of Poly I:C offspring mice [56, 57], and increased mRNA expression of AKT2 and GSK3β was found in the ventral tegmental area of Poly I:C rats [34]. This study found that prenatal Poly I:C challenge led to an increased mRNA expression of AKT1, AKT3, and GSK3β in the DRN; therefore, our findings provided the first evidence in the DRN to support the proposition that alterations in AKT‐GSK3β signaling contribute to schizophrenia pathogenesis. The GSK3β‐mediated Dvl3‐β‐catenin signaling pathway is another GSK3β‐mediated signaling pathway that is related to the effects of antipsychotics [25, 58, 59, 60]. It is consistent that olanzapine and risperidone treatment increased mRNA expression of AKT1, GSK3β, and β‐catenin in the DRN of both healthy and Poly I:C offspring rats, which suggests that the effects of these antipsychotics on GSK3β mRNA expression are shown in DRN. We propose that the AKT‐GSK3β‐mediated Dvl3‐β‐catenin signaling in the DRN also contributes to the therapeutic effects of these antipsychotics.

The cAMP‐PKA pathway is a canonical D2 receptor‐downstream signaling pathway that mediates various cellular responses [61]. For example, D2‐like dopamine autoreceptors control dopamine synthesis, release, and uptake via the PKA pathway [20]. It has been reported previously that 1‐week treatment of haloperidol increased PKA activity in the ventral tegmental area, whereas 10‐week treatment with aripiprazole reduced it [26]. However, this study showed that prenatal Poly I:C exposure did not impact PKA expression in the DRN, while chronic antipsychotic treatment had an overall effect of increasing PKA expression. CREB1 is also a downstream substrate of the PKA pathway [62], and a relationship between CREB1 and the positive symptoms of schizophrenia has been proposed [63, 64]. Previous studies have reported repeatedly that antipsychotics increase CREB1 activity [44, 58, 65]. Consistently, this study showed that both olanzapine and risperidone increased CREB1 expression in the DRN.

## 5 Conclusion

In summary, this study has demonstrated the effects of prenatal Poly I:C‐induced immune activation and antipsychotics treatment on the NMDA and GABA_A_ receptors and PKA‐CREB1, AKT‐GSK3β, and Dvl‐β‐catenin signaling in the DRN of adult rats (Figure 5). Our previous study showed that prenatal Poly I:C exposure caused elevation of NF‐κB and IL‐1β inflammatory signaling via activating toll‐like receptor 3 (TLR3) in the brain of offspring [2]. The main results of this study have shown that prenatal Poly I:C challenge mainly influences the NR1, NR2A, and NR2B subunits of the NMDA receptors, while the antipsychotics exert their effects via influence on the NR2C and NR2D subunits of NMDA receptors. In addition, prenatal Poly I:C exposure mainly influenced the AKT‐GSK3β signaling pathway, while antipsychotics treatment modulated the PKA‐CREB1 and Dvl‐GSK3β‐β‐catenin signaling pathways. Since antipsychotics do not directly bind with NMDA and GABA_A_ receptors, it is possible that antipsychotics modulate these receptors via D2R‐mediated PKA‐CREB1 and Dvl‐GSK3β‐β‐catenin signaling pathways [26, 28, 58, 66]. The exact mechanisms underlying these effects still need further investigation. One limitation is that, due to the very small sample of the DRN nucleus, only mRNA expression was examined in this study. Further studies are necessary to examine the protein levels/protein phosphorylation with Western blot and NMDA/GABAergic neurotransmission by electrophysiology recordings to fully reveal the effects of prenatal Poly I:C exposure and antipsychotic treatment on the DRN. The other limitation in this study is that only female rats have been examined. Since sex differences have been observed in the rodent Poly I:C models [67, 68], further studies are necessary to investigate the effects of prenatal Poly I:C exposure and antipsychotics treatment on the DRN of male rats.

## Author Contributions

Conceptualization: Shiyan Chen, Jiamei Lian, and Chao Deng. Funding acquisition: Chao Deng, Jiamei Lian. Investigation: Shiyan Chen, Jiamei Lian, and Yueqing Su. Formal analysis: Shiyan Chen and Chao Deng. Visualization: Shiyan Chen and Chao Deng. Writing – original draft: Shiyan Chen. Writing – review and editing: Chao Deng, Shiyan Chen, Jiamei Lian, and Yueqing Su. All authors commented on and approved the final draft.

## Ethics Statement

All experiments were approved by the Animal Ethics Committee, University of Wollongong, NSW, Australia (AE 17/12), complying with the National Health and Medical Research Council Australian Code of Practice for the Care and Use of Animals for Scientific Purposes (2013).

## Conflicts of Interest

The authors declare no conflicts of interest.

## Supporting information


**Table S1.** Primers sequence.

## Data Availability

The raw data supporting the conclusions of this article are available from the corresponding authors on reasonable request.
