# Supplementary material for: Differential Effects of Prenatal Poly I:C Exposure and Antipsychotics on NMDA/GABA Receptors and GSK3β‐Mediated Signaling in the Dorsal Raphe Nucleus of Female Rats
Source: Fundam Clin Pharmacol. 2025 Jun 25;39(4):e70033. doi: 10.1111/fcp.70033 (PMC12196557; doi:10.1111/fcp.70033)
Supplement: Supplementary file 1 — Table S1. Primers sequence. [file FCP-39-0-s001.pdf]

Supplementary Table 1 Primers sequence

Markers	Primers	
	Forward primer	Reverse primer
Htr1a	aaagagcaccttcctctg	agagccacaatgaaaaacg
Htr2a	atctgtaggtatatccatgcc	cacaaaagagcctatgagaac
Htr2c	gggcaatatcaataggagtttc	aggacgtagatcgttaagaag
Drd2	caacaatacagaccagaatgag	ggaggacgatgtagattttg
Gabrb3	attgaattttactggcgtgg	ctgaagtatgaagtacccaatg
Gad1	ctactggtttggatatcattgg	ggagaaaatatcccatcacc
Grin1	aaggagaatatcactgaccc	tacttagaagacatcagcacc
Grin2a	gatcaacaattcaaccaacg	agaccacttcacctatcattc
Grin2b	gtttaacaactccgtacctg	tctggaacttcttgtcactc
Akt1	ggggaatatattaaaacctggc	gtcttcatcagctgacattg
Akt2	gagtcctacagaataccagg	aatctctgcaccataaaagc
Akt3	aaaggatccaaataaacgcc	aaggaggtacaagctttttg
Gsk3β	cactcaagaactgtcaagtaac	tccagcattagtatctgagg
Prkaca	ctatattcaggtgacagacttc	ctttgctcaggataatctcg
Prkacb	gtaaagcataaagccactgag	ggattctcttctcattcagag
Creb1	caaacagttcagtcttcctg	agttacactatccacagactc
Ctnnb1	catcaggaaggagctaaaatg	agaatgatgagcttgctttc
Dvl3	actccactatgtctctcaac	gatctcatttacctgcaacag
